# Artificial intelligence in breast cancer imaging: risk stratification, lesion detection and classification, treatment planning and prognosis—a narrative review

**DOI:** 10.37349/etat.2022.00113

**Published:** 2022-12-27

**Authors:** Maurizio Cè, Elena Caloro, Maria E. Pellegrino, Mariachiara Basile, Adriana Sorce, Deborah Fazzini, Giancarlo Oliva, Michaela Cellina

**Affiliations:** 1 Postgraduate School in Diagnostic and Interventional Radiology, University of Milan, 20122 Milan, Italy; 2 Centro Diagnostico Italiano, 20147 Milan, Italy; 3 Department of Radiology, ASST Fatebenefratelli Sacco, 20121 Milan, Italy; University of Campania “L. Vanvitelli”, Italy

**Keywords:** Breast cancer imaging, artificial intelligence, machine learning, computer-aided detection, mammogram, digital breast tomosynthesis, magnetic resonance imaging

## Abstract

The advent of artificial intelligence (AI) represents a real game changer in today’s landscape of breast cancer imaging. Several innovative AI-based tools have been developed and validated in recent years that promise to accelerate the goal of real patient-tailored management. Numerous studies confirm that proper integration of AI into existing clinical workflows could bring significant benefits to women, radiologists, and healthcare systems. The AI-based approach has proved particularly useful for developing new risk prediction models that integrate multi-data streams for planning individualized screening protocols. Furthermore, AI models could help radiologists in the pre-screening and lesion detection phase, increasing diagnostic accuracy, while reducing workload and complications related to overdiagnosis. Radiomics and radiogenomics approaches could extrapolate the so-called imaging signature of the tumor to plan a targeted treatment. The main challenges to the development of AI tools are the huge amounts of high-quality data required to train and validate these models and the need for a multidisciplinary team with solid machine-learning skills. The purpose of this article is to present a summary of the most important AI applications in breast cancer imaging, analyzing possible challenges and new perspectives related to the widespread adoption of these new tools.

## Introduction

Breast cancer is the most common type of cancer in women and the second leading cause of death after lung cancer [1]. With more than 2 million new cases in 2020, it represents a major public health concern for health systems and policymakers [2, 3]. In recent years, radiology has faced exponential growth in artificial intelligence (AI) applications in clinical practice with significant and encouraging results, especially in oncological imaging [4]. Various imaging modalities are currently used in breast cancer imaging: mammography and digital breast tomosynthesis (DBT), ultrasound (US), magnetic resonance (MR), and positron emission tomography (PET), and each could gain significant benefits from AI support [5]. Several studies show that incorporating an AI-based approach into the standard radiological workflow improves breast imaging diagnostic accuracy [6].

AI has multiple applications in breast cancer imaging: 1) risk stratification, in order to achieve individualized screening programs; 2) assisted detection of tumor to increase diagnostic accuracy, reducing the rate of false negatives and false recalls, while improving radiologists’ workload; 3) non-invasive tumor characterization (identification of tumor subtype, evaluation of tumor heterogeneity and microenvironment, etc.) to plan targeted therapy and follow-up; and finally, 4) prognostic/predictive applications regarding response to treatment, risk of relapse and overall survival [7–11].

**Figure 1.**
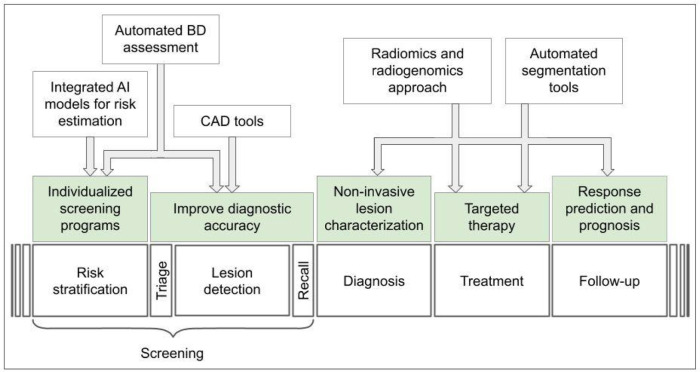
Overview of AI applications in breast cancer imaging. CAD: computer-aided detection; BD: breast density

However, despite the promising prospects, all that glitters is not gold. The integration of AI-powered tools into clinical practice presents new and fascinating challenges for radiologists, which must be considered to successfully address this new frontier [12–14]. An overview of AI applications in breast cancer imaging is presented in Figure 1.

## A quick introduction to AI

AI is a vast field of knowledge. Technology that mimics human intelligence to solve problems is the core of what is collectively called AI. Its outermost shell is machine learning (ML), a term that refers to the automated detection of meaningful patterns in data [15, 16].

**Figure 2.**
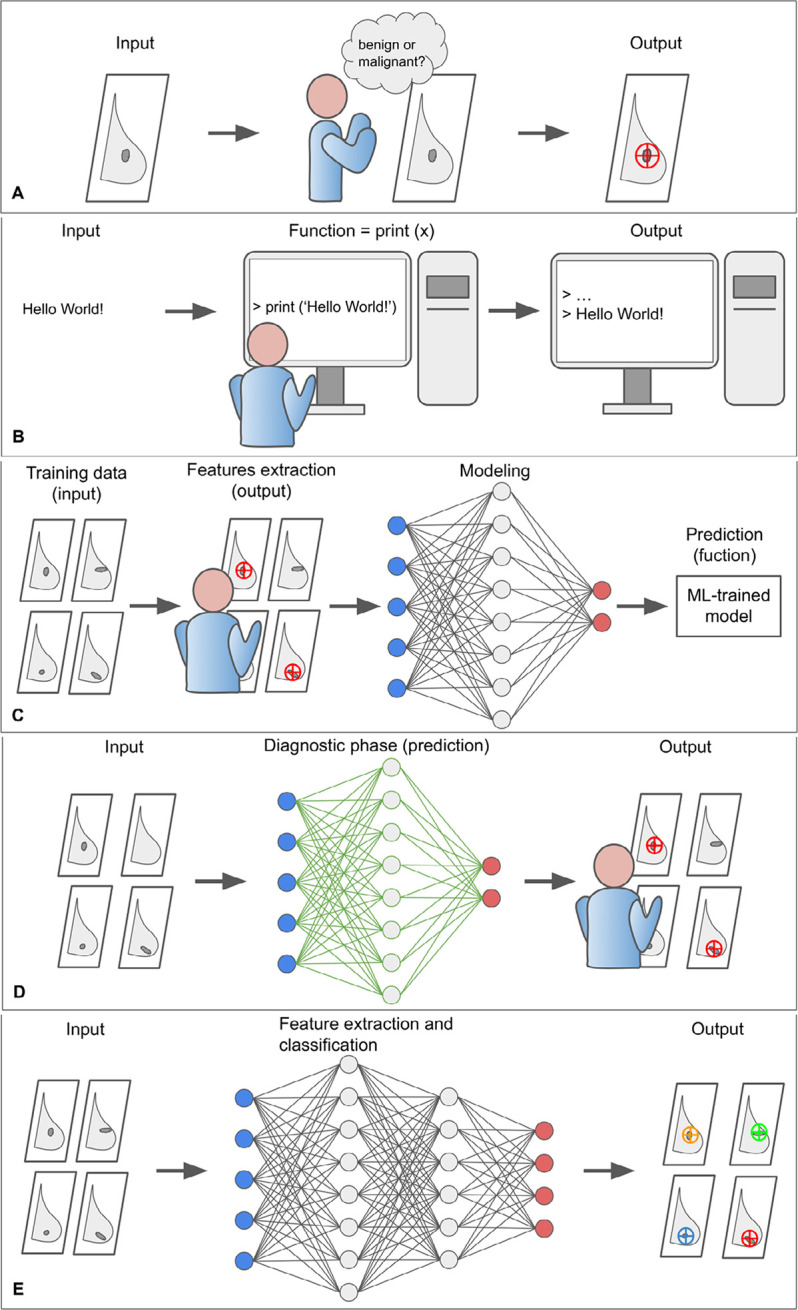
The core concepts of ML paradigm. A. In a normal workflow, the radiologist evaluates the mammogram (input) and determines whether the lesion is benign or malign (output); B. to program a computer to perform a certain operation, it is necessary to specify the function that the machine is to perform. In the example, the function—print (x)—writes the argument of the function to the screen. However, the extremely complex cognitive process of the radiologist cannot be translated into the programming language; C. the classification problem can be addressed through a ML approach. The proposed example is a form of supervised learning that exploit a simple ANN to perform a binary classification task that consists in distinguishing between benign and malignant lesion on digital mammograms. The ML model requires a learning phase in which both raw data and exams already classified by the radiologist are provided. The learning phase of the model includes a training, validation, and testing phase, which are not represented for clarity. The model progressively reduces the uncertainty as it is exposed to more data samples and comes to approximate the “function” normally performed by the radiologis; D. after the ML algorithm has been trained and tested, it can be integrated into clinical practice and used to assist the radiologist in visual assessment; E. DL models exploit particularly complex neural networks to extract and analyse new and intricate image patterns (radiomic features) in large data sets that are usually not accessible to the human operator. ANN: artificial neural network; DL: deep learning

Normally a machine (i.e., a computer) performs operations on input (x) to obtain output (y). To perform such a task, the programmer is required to code the function (*f*) to be computed in the programming language (coding). Since breast cancer screening is challenging and time-consuming, radiologists would benefit from a machine aiding in the classification of breast lesions in mammograms as either benign or malignant. The problem is that, as radiologists are well aware, lesions detection is an extremely complex cognitive task that depends on intrinsic factors, such as the experience and skills of the radiologist, and extrinsic factors, such as the characteristics of the tumor (size, position, morphology, etc.) and the breast (BD, etc.). From a computational point of view, it is not possible to translate into the code what the expert radiologist does in his work routine, mostly automatically (Figure 2).

However, it is possible to address this challenge through an AI-based approach, in which a ML model is trained to infer the desired function by “learning for examples”, in some way simulating the learning modalities of the human brain. More formally, ML attempts to approximate a function *f* by analyzing the input data features that would produce the desired output, satisfying some predefined requirements [17]. It is also expected for the ML model to reduce the uncertainty of the approximation as it is exposed to more data samples. For example, in supervised learning (the simplest form of ML), the learning phase of the model would require a dataset (with at least two subparts, one for training and one for validation) comprising typical examples of inputs (e.g., digital mammograms) and corresponding outputs (e.g., tumor lesions already identified by an experienced radiologist). By feeding the model with enough data, it can learn to infer the relationship that binds them with a desired level of accuracy. After the learning phase, the model should be tested to verify its reliability in a different dataset [16].

From a practical point of view, a ML model consists of a set of rules that map relationships between data. Among different models, ANNs are a biologically-inspired programming paradigm that enables a computer to learn from observational data [18]. ANNs simulate the human brain and consist of several layers of interconnected ‘nodes’ or ‘cells’. Through the training phase, the ANN progressively shapes the weights of its connections toward the implementation of the desired function. After the ANN is trained and tested, it can be applied to a new dataset to analyze and extract information from raw data (Figure 2) [18].

DL is a subdomain of AI that exploits complex ANNs with a large number of intermediate layers, each representing increasing levels of abstraction, to discover intricate patterns in large data sets that go beyond the features that could be extracted by the radiologist [19]. DL-based tools are particularly suitable for medical image analysis, including CAD, disease prediction, image segmentation, image generation, etc. [20]. DL models are built to capture the whole image context and learn complex correlations between local features, resulting in superior performance on image analysis tasks like classifying breast lesions in a screening mammogram as likely malignant or benign [21].

The application of AI tools in the field of diagnostic imaging is the basis of radiomics and radiogenomics approaches. In simple terms, radiomics could be considered synonymous with quantitative imaging [22]. The radiomics approach exploits sophisticated AI-based tools to extract and analyze large quantitative metrics (radiomics features) from medical images, that are typically inaccessible to the human reader, and try to correlate them to clinical features [23–25], in order to improve precision diagnosis and treatment [26, 27]. Radiogenomics could be considered a subset of radiomics applications, aiming to correlate lesion imaging phenotype (“radio”) to the genotype (“genomics”), based on the assumption that phenotype is the expression of genotype [28]. If a specific imaging phenotype is related to a genotype, imaging features analysis could ideally predict cancer molecular patterns and behavior, allowing personalized treatment planning.

## Risk stratification and screening

Early detection of breast cancer is essential for timely management [29]. However, breast cancer screening is a resource-consuming activity in modern radiology departments and radiologists take prime responsibility for image quality and diagnostic interpretation [30]. A position paper published in 2018 in the European Journal of Cancer by Autier and Boniol [29] concluded that “New, effective methods for breast screening are needed, as well as research on risk-based screening strategies”. The importance of establishing more effective and individualized screening programs is well understood considering the limitations of current screening protocols [29]. Small lesions may be difficult to detect by the human eye during routine mammography and present as interval cancers or advanced cancers at the next examination, with a worse prognosis [31]. Although the recent development of DBT, which creates high-resolution three-dimensional (3D) images, increased breast cancer detection while simultaneously reducing false recalls, the impact of false positive results is still relevant, with increased patient anxiety, unnecessary invasive testing, and ultimately increased costs [32–34]. Furthermore, some authors pointed out the methodological limitations of some randomized trials that may have led to exaggerating the effectiveness of screening [29].

AI-based models could find application in the different stages of breast cancer screening: risk stratification, triage or pre-screening phase, exam interpretation, and patients’ recall [35]. Breast cancer screening protocols in Europe generally rely on double-blind reading by two different radiologists, while in the United States a single reader plus CAD is more common [30, 36, 37]. To date, in the United States, the Food and Drug Administration (FDA) has approved several AI tools for application in breast cancer imaging: 10 for BD assessment, 3 for triage or pre-screening phase, 3 for lesion classification, 5 for lesion detection and classification [38], and further authorizations are being evaluated.

### BD evaluation

A woman’s risk of developing breast cancer depends on several factors such as age, personal and family history, and imaging features such as BD [39, 40], which is the radiographic appearance of the absolute amount or percentage of fibro glandular tissue in the breast [41]. High BD is an established major risk factor for breast cancer and a well-known factor limiting the sensitivity of mammographic screening as it may mask cancer [42, 43]. Mammographic evaluation of BD is traditionally held by the expert radiologists’ visual assessment according to breast imaging-reporting and data system (BI-RADS) criteria. However, this approach presents several limitations such as high intra- and inter-observer variability, resulting in low reliability and reproducibility [44–47]. AI-based tools can help both BD quantitative assessment and lesion detection in high-BD mammograms.

Both BI-RADS-based qualitative BD assessment and computer-generated quantitative BD measures have been shown to be associated with breast cancer risk [48, 49]. In a recent study, a validated fully automated system for BD assessment [DenSeeMammo (DSM)] was found positively associated with breast cancer risk and non-inferior to radiologists’ visual assessment [50].

**Figure 3.**
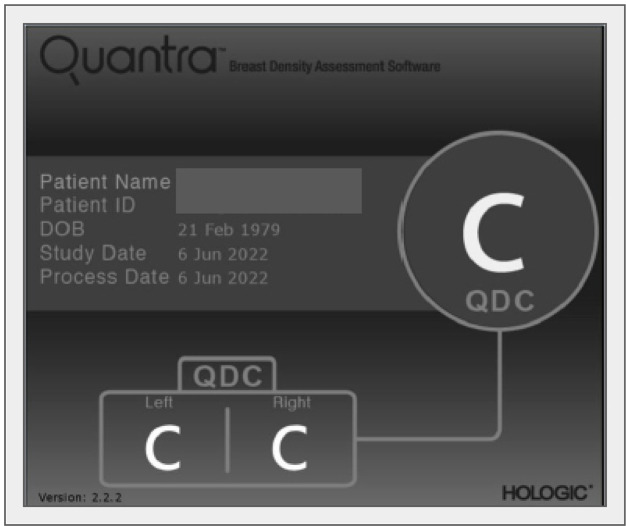
Quantra (HOLOGIC) automated detection of BD according to BI-RADS classification, classified as C in both breasts. The automatic assessment of BD refers to the mammography in Figure 4. QDC: Quantra density category

Several automated or semi-automated software for reproducible assessment of BD have been developed as the Laboratory for Individualized Breast Radiodensity Assessment (LIBRA) [51], Quantra [52], and Volpara [53], and their performance has been evaluated in several studies. For example, BD estimates obtained by Quantra software tend to be lower than visual estimates, however, they correlate well with the BI-RADS BD categories visually assigned to the mammograms (Figure 3) [54]. According to Ekpo et al. [55], Quantra is a poor predictor of BI-RADS assessment on a four-grade scale, but well reproduces BI-RADS rating on a two-grade scale.

Deep-LIBRA, an AI system recently validated on a multi-racial, multi-institutional dataset of 15,661 images using convolutional neural network architectures, demonstrated a strong agreement of BD estimates between AI and gold-standard assessment by an expert reader [56].

Advanced DL algorithms enable the extraction of imaging texture features of breast tissue other than BD from standard screening mammographies, such as energy, contrast, correlation, and so on, which can be used to predict the risk of developing breast cancer [57]. A study by Arefan et al. [58] evaluated the feasibility and performance of two DL methods (denoted GoogLeNet and GoogLeNet-LDA) in a case-control setting: it demonstrated that both exhibited superior performance in predicting breast cancer risk than the percentage of BD alone.

### Risk stratification

Several individualized breast cancer risk prediction models have been proposed [59–61] and the effectiveness of individualized *versus* universal screening programs has been investigated in numerous randomized trials such as the Tailored Breast Screening Trial (TBST) [62], women informed to screen depending on measures of risk (WISDOM) [63], and My Personalized Breast Screening (MyPeBS) [64].

One of the most popular risk prediction models is the International Breast Intervention Study (IBIS) model, or Tyrer-Cuzick (TC) model, a scoring system guiding breast cancer screening and prevention by accounting for age, genotype, family history of breast cancer, age at menarche and at first birth, menopausal status, atypical hyperplasia, lobular carcinoma *in situ*, height, and body mass index (BMI) [60]. Despite its widespread use, however, IBIS/TC model demonstrated limited accuracy in some high-risk patient populations [65].

AI can help integrate imaging features into predictive risk models increasing accuracy. For example, a study by Yala et al. [7] evaluated the performance of a hybrid DL model considering both traditional risk factors and mammograms, in comparison with the IBIS/TC model alone: the hybrid model placed 31% of patients in the top risk category, compared with 18% identified by the IBIS/TC model, and was able to identify the features associated with long-term risk beyond early detection of the disease.

### Pre-screening

AI tools are giving promising results in the pre-screening or triage phase to identify likely negative mammograms from those that need further evaluation by an experienced radiologist, with a significant reduction in the workload. A study by Rodriguez-Ruiz et al. [66] evaluated the performance of a new AI system to preliminary rule out mammograms with a low probability of cancer before the radiologist assessment. The balance between workload and diagnostic performance depended on the risk threshold chosen: with a low-risk threshold, they obtained an exclusion rate of only 1% of exams with cancer with a significant reduction in the workload (17%) as well [66]. Similar results have been obtained by Dembrower et al. [67] in a study proving that AI-assisted mammography analysis could reduce radiologist workload and increase cancer detection.

A recent study by Balta and colleagues [68] evaluated the performance of a single *versus* double reading screening program in pre-assessed exams by an AI system that assigns a score from 1 to 10 on each screening exam indicating the likelihood of cancer. It was found that the use of single reading instead of double reading in tests with a low probability of cancer (score 1 to 7), led to unaffected detection (no screen-detected cancers were missed and even the AI detection score was low the single-reader would recall the exam for double check), with a recall rate down 11.8% of the workload down 32.6% [68].

### Lesion detection

**Figure 4.**
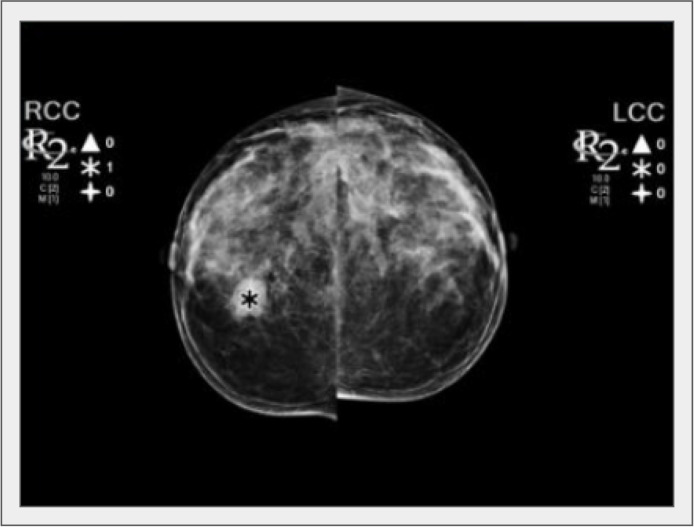
A nodule was detected by the CAD tool in the right breast (asterisk). LCC: left craniocaudal view; RCC: right craniocaudal view

Assisting the radiologist in improving lesion detection is one of the primary goals of AI-based tools (Figure 4). After the United States FDA approved CAD for mammography in 1998, several studies evaluated the accuracy of CAD software in mammography screening, with initially controversial results. Unfortunately, some authors observed that CAD systems adversely affect some radiologists’ performance and increase recall rates [69]. Against an estimated cost of over $400 million annually, a study by Lehman et al. [37] found no evidence that CAD applied to digital mammography significantly improves screening performance.

However, before 2020, AI algorithms were developed using small mammography data sets collected by one or two institutions, limiting verification of the robustness of those algorithms [70], instead, numerous evidence has emerged in the last two years that supports the use of AI in mammography screening as a complementary diagnostic tool, both in single and double reading settings, with significant performance gains.

In an international crowdsourced challenge, Schaffter et al. [71] underscored the potential of using ML methods to enhance mammography screening interpretation: even if no single AI algorithm outperformed radiologists, an ensemble of AI algorithms combined with radiologist assessment in a single-reader screening environment showed to improved overall accuracy.

A European study conducted on screening mammograms of 429 consecutive women diagnosed with interval cancer, positively addressed the question of whether a DL-based AI system could reduce interval cancer in mammography screening, without additional screening modalities [72].

Recent retrospective studies based on a large population have confirmed the usefulness of AI-based approaches. In a study conducted in Europe including 122,969 mammography examinations from 47,877 women, AI showed promising results according to the proportion of screen-detected cancers detected by AI at different preselected thresholds [73]. In another large retrospective study, a DL algorithm was developed and validated with 170,230 mammography examinations collected from five institutions in South Korea, the United States, and the UK, showing a better diagnostic performance in breast cancer detection compared with radiologists [70].

In a study by Kim et al. [74] the use of an AI-based system demonstrated significant added value in detecting mammographically occult breast cancers: 97.5% were found in heterogeneous or extremely dense breasts, 52.5% were asymptomatic, 86.5% were invasive, and 29.7% already had axillary lymph node metastases.

Many studies conducted to date present several limitations: most of them are small and retrospective in nature. Therefore, further prospective trials are required. The milestone MASAI study, a randomized controlled trial, still ongoing, with 100,000 participants, is aiming to assess whether AI can improve the efficacy of mammography screening with the application of AI in the main phases of mammographic screening, having interval cancer as the principal endpoint [75].

Unlike traditional mammography, DBT operates in a 3D domain and differs in acquisition time, acquisition angle, and the number of images. For these reasons, the application of AI models to DBT represents a considerable challenge. Several studies have evaluated the performance of DL-based algorithms in identifying suspicious masses, architectural distortions, and microcalcifications in DBT images [76, 77]. In a recent study, van Winkel et al. [78] evaluated the impact of AI on accuracy and reading time in DBT interpretation, demonstrating that radiologists improved their performances when using an AI-based support system. Another study by Conant et al. [79] compared the performance of 24 radiologists (13 of whom were breast subspecialists) in reading 260 DBT examinations (including 65 cancer cases), both with and without AI support. The AI-based tool was found to improve radiologist performance in the detection of malignant lesions, with a reduction in recall rate and reading time [79].

Still, the validation of these AI systems requires huge case series. Buda et al. [80] curated and annotated a data set of DBT studies including 22,032 reconstructed DBT volumes from 5,060 patients and made this data set publicly available at the Cancer Imaging Archive [81] a public data hosting service for medical images of various modalities, to develop and test a DL algorithm for breast cancer detection.

Concerning MR imaging (MRI), actually, the only FDA-approved CAD is QuantX™ from Qlarity Imaging. QuantX™ is indicated for the assessment and characterization of breast abnormalities from MRI data in patients presenting for high-risk screening, diagnostic imaging workup, or evaluation of the extent of known disease. In a retrospective, clinical reader study, the application of this tool improved radiologists’ performance in the task of differentiating benign and malignant MRI breast lesions, showing an increase in the average area under the curve (AUC) of all readers from 0.71 to 0.76 (*P* = 0.04) when using the AI system [82].

## Lesion characterization

Breast cancer is a clinically and biologically heterogeneous disease, with several recognized histotypes and molecular subtypes [83]. Subtype discrimination is essential for planning targeted therapy. Currently, four molecular subtypes have been identified: 1) luminal A; 2) luminal B; 3) human epidermal growth factor receptor 2 (HER2)-enriched; and 4) basal-like, which have critical differences in incidence, response to treatment, disease progression, survival, and imaging characteristics [84]. High-throughput discrimination of breast cancer subtypes and inter- and intra-tumoral heterogeneity is essential to set up targeted therapies [85]. However, this could only be partially accounted for by current diagnostic tools. In this landscape, valuable help could come from AI through its radiomics and radiogenomics approaches, representing a considerable boost toward personalized medicine [86–89].

Nowadays, tumor genotype and molecular characterization require invasive techniques like surgery or biopsy to collect tissue samples. In addition to common complications like pain, bleeding, hematoma, and breast implant damage, the most important bias in biopsy is the missed diagnosis due to insufficient material collected, especially in small lesions [90]. Moreover, since the tissue sample is a small portion of a heterogeneous lesion, molecular analysis results may not be accurate for the entire lesion [91] and large-scale genome-based cancer characterization is not habitually performed due to high costs, time consumption, and technical complexity [91]. Finally, the tumor genome may change over time making treatment less effective, however, the biopsy is not the ideal method for tracking tumor evolution.

By combining multimodality imaging data, AI tools could complement traditional histological assessment by overcoming some of the limitations of biopsy. First of all, AI image analysis software could evaluate the whole 3D tumor lesion and its surrounding microenvironment [92]. Moreover, AI makes it possible to assess multiple lesions and track them at different time points, allowing physicians to adapt targeted therapy over time [93].

To date, some early attempts to match genomic and imaging data have been made through the creation of two archives: The Cancer Genome Atlas (TCGA) which collects several genomic and clinical cancer biomarkers, and The Cancer Imaging Archive (TCIA) containing corresponding imaging data, with the limit of different protocols, sometimes used [24].

In the last decade, radiomics analyses have been applied to mammography and DBT, breast US, and MRI, with promising results, and AI tools have been successfully applied to extract radiomic features [88, 94]. In 2019, a radiomics approach was applied in a retrospective study on 331 cancer cases aiming to automatically extract radiomics features from digital mammograms. Both qualitative and agnostic features have been evaluated and four of them showed a statistically significant (*P* < 0.05) difference: concavity, correlation, roundness, and gray mean (calculated from the histogram of tumor voxel intensities). More specifically, triple-negative samples have shown a smaller concavity, a larger roundness, and a major gray mean than HER2-enhanced and luminal samples [95].

In some pioneering studies, AI-assisted tools have been tested and evaluated in US imaging, in order to double-check radiologist interpretation and eventually reduce unnecessary biopsies. In a study by Wang et al. [96] the performance of two AI-based downgrading stratification methods was evaluated using histopathological results as the reference standards. Stratification method A was used to downgrade only if the assessments of both orthogonal sections were possibly benign and it showed promising results: forty-three lesions diagnosed as BI-RADS category 4A by conventional US received a hypothetical AI-based downgrade, reducing the biopsy rate from 100% to 67.4% (*P* < 0.001) without losing malignancies [96].

Several studies evaluated the performance of AI tools in the analysis of dynamic contrast-enhanced (DCE) MR (DCE-MR) and diffusion-weighted imaging (DWI) data. When using a radiogenomics approach, researchers are interested in investigating the correlation between DCE-MR or DWI characteristics such as tumor size, shape, morphology, and genomic features, such as protein expression and mutations [97, 98]. For example, a study by Zhu et al. [98] found that transcriptional activities of various genetic pathways were positively associated with tumor size, blurred margins, and irregular tumor shape.

Several studies have focused on evaluating the effectiveness of a radiomics-based approach in distinguishing between malignant and benign lesions, exploiting the possibility of identifying and quantifying features otherwise difficult to be recognized by the human reader [22]. Entropy is an important imaging feature in tumor lesions reflecting the tumoral heterogeneity and its vascular status [99]. In an MRI-based radiomics study, an advanced ML tool was evaluated and the entropy value was found to be a useful parameter to distinguish malignant lesions compared to benign lesions [100]. Another study based on DCE-MR data tried the additive benefit of a set of quantitative features such as irregularity and entropy over maximum linear size alone to differentiate luminal A breast cancers from benign breast lesions, with promising results [101].

The correlation between MR features and molecular breast cancer subtypes has been investigated using traditional and radiomics-based approaches [24, 102]. Leithner et al. [103] evaluated the performance of an AI-based open-source software (MaZda 4.6) in the extraction of radiomics features aiming to assess breast cancer molecular subtypes. The discrimination between luminal A and triple negative cancers had the best statistical results, with an overall median AUC of 0.8 and median accuracies of 74% in the training dataset and 68.2% in the validation dataset [103]. A study by Li et al. [104] evaluated the performance of a classifier model for molecular subtyping: statistically significant associations were found between tumor phenotypes and receptor status, with aggressive cancers that tend to be larger in size with more heterogeneity in their contrast enhancement.

In another study, Yeh et al. [105] performed a radiomic analysis of different MR features to study the underlying activity of multiple molecular pathways that regulate replication, proliferation, apoptosis, immune system, and extracellular signaling. The results showed that tumors with upregulation of immune signaling pathways such as T-cell receptor and chemokine signaling, as well as extracellular signaling pathways, are associated with typical imaging features. The results suggest the possibility to infer the most immunologically active tumors and predict the effectiveness of immunological therapies [105]. Couture et al. [8] trained a DL image analysis tool on a data set of 571 breast tumors to create an image-based classifier assessing tumor grade, estrogen receptor (ER) status, prediction analysis of microarray 50 (PAM50) profile, histologic subtype, and risk of recurrence score. DL-based image analysis was able to distinguish low-intermediate *versus* high tumor grade (82% accuracy), HER status (84% accuracy), basal-like *versus* non-basal-like (77% accuracy), ductal *versus* lobular (94% accuracy), high *versus* the low-medium risk of recurrence score (75% accuracy) [8].

## Whole-breast and tumor segmentation

Tumor segmentation is an important task in oncological imaging [106]. It consists of image analysis and delimitation of the regions of interest (ROI) comprising the tumor from a 2D or 3D acquisition [107]. Manual segmentation is a time-consuming task, affected by a high degree of inter-reader variability due to the limitations of the human reader to solve the lesion-background relationship unambiguously, especially in lesions with blurred margins or in the high-density breast. AI-assisted tools based on DL algorithms can reduce segmentation time and significantly increase reproducibility and efficiency [106]. In breast cancer imaging, it could be potentially useful in different tasks such as treatment planning, and lesion follow-up, but also prognostic and predictive evaluations (Figure 1). Furthermore, tumor segmentation is an essential step in the radiomic workflow, necessary for the extraction of radiomic features [17, 22].

Jiang et al. [108] developed a fully automated algorithm for accurate segmentation of the whole breast using 3D fat-suppressed DCE-MR images and demonstrated a good overlap with manual segmentation. Zhang et al. [109] tested two DL models (UNet and SegNet) as segmentation methods in diffusion-weighted MR images and compared them to manual segmentation used as the reference standard. The study demonstrates that the DL models could achieve promising segmentation results to help computer-aided quantitative analyses of breast DWI images [109].

The assessment of normal breast tissue is essential to improve lesion detection and involves some segmentation tasks. In the first instance, the total amount of fibroglandular tissue is a well-known breast cancer risk factor (see also BD evaluation) [110]. In a study by Huo et al. [111], a DL algorithm was tested on a dataset of 100 breast DCE-MR previously assessed by expert radiologists according to BI-RADS criteria, showing a high correlation coefficient (0.981) between the breast densities obtained with the DL-based segmentation and the manual assessment.

Furthermore, the level of background parenchymal enhancement (BPE) in DCE-MR images is considered an independent marker of breast cancer risk and breast cancer treatment outcomes [112]. In a case-control study, Saha et al. [113] evaluated an AI-based model to assess quantitative measures of BPE in 133 women at high risk for developing breast cancer. AI-extracted BPE offered a more precise and reproducible measuring than humans and may potentially be used to further stratify risk in patients undergoing high-risk screening MRI [113].

Fully automated methods for assessing both fibroglandular tissue and BPE were developed and validated. Fibroglandular tissue was segmented in T1-weighted, nonfat-saturated MRI, and then propagates this segmentation to DCE-MR to quantify BPE within the segmented fibroglandular areas [112]. High spatial correspondence was observed between the automatic and manual fibroglandular tissue segmentation and both fibroglandular tissue and BPE quantifications indicated a high correlation between automatic and manual segmentations. However, a poor correlation was found between segmentation and clinical rating [114]. Similar studies have been proposed by Ma et al. [110] and Ha et al. [115].

An AI-assisted lesion segmentation tool has been applied to automated whole breast US by Lee et al. [116], aiming to overcome the amount of speckle noise and the low contrast of the lesion boundaries typical of this ultrasonic image technique. The ground truth was assessed by two experienced radiologists in breast US, the proposed method demonstrated high accuracy in processing automated whole breast US images, segmenting lesions, calculating lesion volumes, and visualizing lesions to facilitate observation by physicians [116].

A better 3D reconstruction and a more precise margin evaluation can improve preoperative planning. In patients receiving radiotherapy, proper target delineation can reduce radiation doses to the nearby normal organs at risk. The feasibility of a DL-based auto-segmentation tool was demonstrated by Chung et al. [117], the correlation between the auto-segmented and manually segmented contours was acceptable and the differences in dosimetric parameters were minimal. Recently, Byun et al. [118] assessed the performance of another DL auto-contouring system with a group of experts. Manual contours, corrected auto-contours, and auto-contours were compared. Inter-physician variations among the experts were reduced in corrected auto contours, compared to variations in manual contours; furthermore, the DL tool revealed good user satisfaction [118].

While AI methods are unlikely to replace the work of radiation oncologists, they could be a useful tool with excellent potential for assisting radiation oncologists in the future, improving the quality of breast radiotherapy, and reducing inter-reader variability in clinical practice.

## Prognosis

One of the most important AI applications in breast cancer imaging is the development of new predictive and prognostic models based on a radiomic approach [23, 88, 119]. Traditionally prognostic factors include age, number of positive axillary lymph nodes, tumor size, tumor grade, lymphatic and vascular invasion, and immunohistochemical biomarkers such as ER/progesterone receptor (PR) status, HER2, and Ki-67 [120, 121]. Additional factors include grade, presence of lymphovascular invasion, age, and ethnicity. Certain biologic factors, including ER/PR and HER2/neu, are both prognostic and predictive [121].

AI enables the integration of quantitative radiological data from various imaging modalities with patient clinical data (e.g., family history, molecular and genomic data) with the aim of improving the predictions of relevant clinical endpoints such as disease-free survival (DFS), progression-free survival, complete response, and others [23, 122]. Furthermore, radiomics-based approach allows the potential identification of new imaging biomarkers [88].

MR-based AI models have been developed to predict response to neoadjuvant chemotherapy at an early stage or even prior to the beginning of the treatments. These AI tools could be used to avoid the administration of ineffective and potentially toxic therapies, as well as to expedite surgery in patients who would not benefit from neoadjuvant chemotherapy. Furthermore, surgery may be avoided in patients who have a pathologic complete response after neoadjuvant chemotherapy [123].

ML was tested in the early prediction of complete response to neoadjuvant chemotherapy and survival outcomes in breast cancer patients through the analysis of multiparametric MR examinations performed on a 3 Tesla equipment [124]. There were 23 features extracted for each lesion: qualitative T2-weighted and DCE-MRI features according to BI-RADS, quantitative pharmacokinetic DCE features (mean plasma flow, volume distribution, mean transit time), and DWI apparent diffusion coefficient values. To apply ML to multiparametric MR examinations, 8 classifiers including linear support vector machine, linear discriminant analysis, logistic regression, random forests, stochastic gradient descent, decision tree, adaptive boosting, and extreme gradient boosting (XGBoost) were applied to rank the features. Histopathologic residual cancer burden class, recurrence-free survival, and disease-specific survival were used as the standards of reference. The study demonstrated the high accuracy of the ML model in predicting both residual cancer burden (AUC, 0.86) and disease-specific survival (AUC, 0.83).

When compared to other classifiers, the XGBoost achieved the most stable performance with high accuracy. Changes in lesion size, complete pattern of shrinkage, mean transit time on DCE-MRI, minimum apparent diffusion coefficient on DWI, and peritumoral edema on T2-weighted imaging, were the most important features for predicting residual cancer burden. On the other hand, volume distribution, mean plasma flow and mean transit time, DCE-MRI lesion size, and minimum, maximum, and mean ADC with DWI were the most important features for predicting recurrence-free survival. On DCE-MRI, the most important features for predicting disease-specific survival were lesion size, volume distribution, and mean plasma flow, as well as maximum ADC with DWI. In a multicenter study, Liu et al. [125] obtained similar results using a radiomics multiparametric model with four radiomic signatures.

Bitencourt et al. [126] used AI in conjunction with clinical variables to assess the complete pathologic response after neoadjuvant chemotherapy in overexpressing HER2 breast cancer and found that it was 83.9% accurate. Another study on HER2-positive cancer responses was conducted by Braman et al. [127], who analyzed intra and peritumoral features. In both validation cohorts, their model was able to identify the HER2 breast cancer subtype with an AUC of 0.89 and predict neoadjuvant chemotherapy response to HER2-targeted therapy (AUC of 0.80 and 0.69, respectively). Cain et al. [128] used pretreatment MR of 288 patients to predict response to neoadjuvant chemotherapy developing a multivariate ML-based model. Twelve features were chosen, six from the tumor alone, five from the fibroglandular tissue alone, and one from both. The “change in variance of uptake”, a tumor-based feature that quantifies the change in variance of tumor uptake in two consecutive time points, was found to be the most relevant feature [128].

Sutton et al. [129] in a study on 273 women with 278 invasive breast cancers, developed a model that combined MRI-extracted radiomics features with molecular subtypes to identify pathologic complete response post neoadjuvant treatment, which showed an AUC of 0.78 on the test set.

The radiomics approach has the potential to extract quantitative imaging biomarkers that can be used to predict DFS. In 620 patients with invasive breast cancer, Xiong et al. [130] evaluated the added value of the US radiomics signature. Independent of clinicopathological predictors, the radiomics signature was significantly associated with DFS and outperformed the clinicopathological nomogram. Other authors have confirmed the usefulness of the radiogenomic approach in the subgroup of patients with triple-negative breast cancer [131].

A recent study evaluated preoperative MRI of 294 patients with invasive breast cancer and concluded that radiomics nomograms could significantly improve DFS prediction individualization [132]. A study that included patients with HER2-positive invasive breast cancer came to similar conclusions [133].

Cancer recurrence prediction is another relevant clinical issue in patient management and AI-based MRI models have demonstrated their potential in recurrence prediction [134, 135]. For example, Ha et al. [136] used a CNN to predict Oncotype Dx recurrence score, using MRI datasets for the distinction of the low, medium, and high-risk patients, with an overall accuracy of 81% in a three-class prediction with a specificity of 90%, a sensitivity of 60%, and AUC of 0.92.

Kim et al. [137] collected data on 679 breast cancer surgery patients, including histological grade, tumor size, number of metastatic lymph nodes, ER, lymphovascular invasion, local invasion of the tumor, and number of tumors, to develop AI-based models to estimate the risk of recurrence. One of the models obtained demonstrated high sensitivity (0.89), specificity (0.73), positive predictive values (0.75), and negative predictive values (0.89). The authors were adamant that grouping patients into high-risk and low-risk categories help with treatment and follow-up planning.

Finally, studies demonstrated that a radiomics-based AI model was able to predict the presence of sentinel or axillary lymph node metastases [138, 139]. For example, Dietzel et al. [140] demonstrated that a breast MRI-based ANN can predict axillary lymph node metastasis with an AUC of 0.74.

## Challenges and perspectives

Publications on AI in medicine have increased exponentially in recent years, demonstrating the great interest in these applications. However, despite the rapidly growing hype, some challenges initially frustrated its clinical application.

In the first instance, clinicians are not always adequately equipped to deal with this paradigm shift. Radiologists in particular feel a pressing threat to their professional expertise coming from these new tools [12]. However, the applications of AI in medical imaging could open up new scenarios allowing radiologists to perform more value-added tasks while avoiding repetitive and time-consuming ones playing a pivotal role in multidisciplinary clinical teams [12, 14].

It is unlikely that radiologists will be replaced because their work includes not only image interpretation [141]. In breast imaging, in particular, radiologists have a close relationship with the patient that includes diagnosis communication, the outline of the diagnostic path based on patient values and preferences, the overall medical judgment integrating different types of clinical information that go beyond imaging, interventional procedures interventional procedure and quality insurance [12, 14]. To take full advantage of the opportunities presented by a quantitative imaging approach radiologists must develop new cross-disciplinary and multidisciplinary skills. This knowledge can hardly be managed by a single radiologist, especially in the research field, which is why multidisciplinary teamwork is becoming more and more important. This fact can be a barrier for small research groups and institutions that lack the financial resources to recruit non-medical professionals to dedicate exclusively to research and support activity in AI.

Other crucial topics concern technical and methodological issues. The need for large amounts of data is a limitation to the development of these models which should be trained, validated, and tested on big data sets. In recent years, the growing popularity of open-source image repositories has partially addressed these issues and encouraged the development of AI-based tools through data sharing, open-access education, and collaborative research [142].

The lack of reproducibility and validation of radiomic studies is considered to be a major challenge in this field. In order to address this problem, the image biomarker standardization initiative (IBSI) was built as an independent international collaboration that works towards standardizing the extraction of image biomarkers from acquired imaging for the purpose of high-throughput quantitative image analysis [143, 144].

Finally, even validated AI tools with high performance would likely fail to significantly improve patient management if they were not well integrated into existing clinical workflows. Further studies are needed to verify the actual impact that the application of these tools has on patient management and outcomes. Such a type of translational research requires close collaboration between breast radiologists, physicists, statisticians, and suppliers of AI tools. For this reason, creating a shared language and methods is an even more pressing need to face these challenges.

## Conclusions

The application of AI in breast imaging has rapidly evolved from the research phase to clinical application. Softwares for automatic lesion detection, macroscopic classification (e.g., malignant/benign), and BD assessment are progressively integrated into the workflows of radiology departments, albeit with some latency in Europe compared to the United States, where these tools are already part of the clinical routine. Furthermore, AI-based algorithms for advanced tasks such as risk stratification, non-invasive molecular characterization of lesions, treatment response prediction, and prognosis are showing promising results, but need to be refined and validated in large case series. It is also essential to understand their real-world impact and how to properly integrate them into the clinical workflow. However, the widespread adoption of these techniques will have a great impact on patient management, moving toward personalized medicine.

## Abbreviations

3D: three-dimensional
AI: artificial intelligence
ANN: artificial neural network
AUC: area under the curve
BD: breast density
BI-RADS: breast imaging-reporting and data system
BPE: background parenchymal enhancement
CAD: computer-aided detection
DBT: digital breast tomosynthesis
DCE-MR: dynamic contrast-enhanced magnetic resonance
DFS: disease-free survival
DL: deep learning
DWI: diffusion-weighted imaging
ER: estrogen receptor
FDA: Food and Drug Administration
HER2: human epidermal growth factor receptor 2
IBIS: International Breast Intervention Study
ML: machine learning
MR: magnetic resonance
MRI: magnetic resonance imaging
TC: Tyrer-Cuzick
US: ultrasound

## Declarations

### Copyright

© The Author(s) 2022.
